# Metagenomics Reveals That Proper Placement After Long-Distance Transportation Significantly Affects Calf Nasopharyngeal Microbiota and Is Critical for the Prevention of Respiratory Diseases

**DOI:** 10.3389/fmicb.2021.700704

**Published:** 2021-09-20

**Authors:** Yaocheng Cui, Jiancheng Qi, Dongjie Cai, Jing Fang, Yue Xie, Hongrui Guo, Shiyi Chen, Xiaoping Ma, Liping Gou, Hengmin Cui, Yi Geng, Gang Ye, Zhijun Zhong, Zhihua Ren, Yanchun Hu, Ya Wang, Junliang Deng, Shuming Yu, Suizhong Cao, Huawei Zou, Zhisheng Wang, Zhicai Zuo

**Affiliations:** ^1^Key Laboratory of Animal Disease and Human Health of Sichuan Province, College of Veterinary Medicine, Sichuan Agricultural University, Chengdu, China; ^2^College of Animal Science and Technology, Sichuan Agricultural University, Chengdu, China; ^3^Animal Nutrition Institute, Sichuan Agricultural University, Chengdu, China

**Keywords:** beef calf, transportation, nasopharyngeal microbial community, metagenomic shot-gun sequencing, adaptive placement, microbiota

## Abstract

Transportation is an inevitable phase for the cattle industry, and its effect on the respiratory system of transported cattle remains controversial. To reveal cattle’s nasopharyngeal microbiota dynamics, we tracked a batch of beef calves purchased from an auction market, transported to a farm by vehicle within 3 days, and adaptively fed for 7 days. Before and after the transport and after the placement, a total of 18 nasopharyngeal mucosal samples were collected, and microbial profiles were obtained using a metagenomic shotgun sequencing approach. The diversity, composition, structure, and function of the microbiota were collected at each time point, and their difference was analyzed. The results showed that, before the transportation, there were a great abundance of potential bovine respiratory disease (BRD)-related pathogens, and the transportation did not significantly change their abundance. After the transportation, 7 days of placement significantly decreased the risk of BRD by decreasing the abundance of potential BRD-related pathogens even if the diversity was decreased. We also discussed the controversial results of transportation’s effect in previous works and the decrease in diversity induced by placement. Our work provided more accurate information about the effect of transportation and the followed placement on the calf nasopharyngeal microbial community, indicated the importance of adaptive placement after long-distance transport, and is helpful to prevent BRD induced by transportation stress.

## Introduction

The homeostasis of microflora colonized in the respiratory tract plays a vital role in maintaining airway health (Man et al., 2017). Emerging evidence indicated that, in cattle, the lung microbial community was more familiar with those in the nasopharynx than other upper respiratory niches (Mcmullen et al., 2020). Losing the balance of beef cattle’s nasopharyngeal microorganisms contributes to the morbidity and mortality associated with bovine respiratory diseases (BRDs) (Holman et al., 2015; Zeineldin et al., 2017). Hence, the nasopharynx microbial community is regarded as the respiratory health situation indicator, especially BRD (Qi et al., 2021; Zeineldin et al., 2020).

The composition and diversity of cattle’s nasopharynx microbial community are affected by many factors, including diet (Qi et al., 2021), vaccination and environment (Mcmullen et al., 2018), and transport (Pratelli et al., 2021). In China and North America, long-distance transport was an inevitable phase for the cattle industry (Cirone et al., 2019). Besides affecting the nervous, endocrine, immune, and energy supply systems (Van Engen and Coetzee, 2018), the latest research found that transportation also affected cattle’s blood transcriptome, indicating that transport affected B cells’ activity (Zhao et al., 2021). It is not surprising that transport was widely considered an intense BRD trigger (Earley et al., 2017; Zeineldin et al., 2019). Hence, exploring the effect of transport on the cattle’s nasopharynx microbial community is of great value in cattle farming.

To date, there is limited research regarding the cattle’s nasopharynx microbial community during transportation. Utilizing 16S rRNA sequencing technology, Holman et al. (2017) found that 2 days of transportation significantly decreased the diversity and richness of cattle’s nasopharynx microbial community, and the decrease of diversity and richness recovered after a few days of adaptive feeding. Their team also implied a connection between the diversity of transported cattle’s nasopharynx microbial community, especially lactic acid-producing bacteria and BRD (Amat et al., 2019). It is accepted that the susceptibility of BRD significantly increased in the first week after transport (Holman et al., 2017; Zeineldin et al., 2017), and selenium-biofortified alfalfa hay treatment in this period was found to benefit the recovery of microbial diversity and to help prevent BRD (Hall et al., 2017). However, these limited references are not enough to draw a convincing conclusion.

Besides the lack of references, technologies such as traditional bacterial culture technology and 16S rRNA sequencing also weaken the drawn conclusion. Although they are widely used to study respiratory tract microflora, they have their disadvantages. For example, many important bacteria are uncultivable, and the depth of 16S rRNA sequencing is not enough to identify microorganisms at the species level. In the past years, with the progress in the next-generation sequencing technologies, metagenomics-based studies are being widely applied to determine the composition of various microbiomes and analyze their functions at the DNA and RNA levels (Wang et al., 2015; Gilbert et al., 2016; Qi et al., 2021).

Therefore, to access the longitudinal dynamics of the transported beef calf nasopharynx microbial community before and after long-distance transport and the following adaptive placement, we collected the nasopharynx swab samples of 18 healthy beef calf before and after 3 days of transportation and 1 week after transportation. The nasopharyngeal microbiome profiles were obtained by the Whole Genome Shotgun (WGS) sequencing approach, and the dynamics in composition, diversity, structure, and function of these nasopharynx microbial communities were analyzed. As far as we know, this is the first time that a metagenomic shotgun sequencing approach is used in studying the dynamics of the nasopharynx microbial community during long-distance transportation. Our study will help understand the effect of transport and the followed adaptive placement on calf respiratory tract microflora and provide a reference for preventing respiratory system diseases related to transportation.

## Materials and Methods

All protocols used in the study referred to the Guidelines for the Care and Use of Laboratory Animals (National Institutes of Health, Bethesda, MD, United States) and were approved by the Ethics Committee of Sichuan Agricultural University (Chengdu, China). All methods were carried out following relevant guidelines and regulations.

### Animals

In the auction market of Qiqihaer, 112 clinical healthy male Simmental calves (0.5 years old and 206 ± 9 kg) were purchased and then transported to a farm in Guangan for fattening by vehicles through the expressway. The criteria of clinical healthy are based on previous studies (Mcguirk and Peek, 2014), and the specific criteria are as follows: the mental outlook and appetite for food and water must be good; the shape of feces and the color of urine must be normal; and the secretion of eyes, mouth, and nose must be normal. The distance was approximately 3,000 km, and the transportation took three consecutive days, and all the calves were restricted from eating and drinking. As soon as the vehicles arrived at the farm, the calves were unloaded and then housed in a single pen for adaptive feeding for 1 week. All the calves were supplied with 3 L of drinking water, which contained brown sugar (0.5 kg/10 L) and ginger (0.3 kg/10 L) for the first three consecutive days and with regular drinking water for the other 4 days. The fodders were free to access in all 7 days. All these adaptive placement measures are conducted according to the farm management manual.

### Sample Collection, DNA Extraction, Sequencing, and Quality Control

Eighteen calves were randomly selected and marked for sampling. Nasopharynx swabs of each selected calf were collected at three respective points: 6 h before loading, named group A, unloading, named group B, and the last day of the adaptive feeding, named group C. The nasopharyngeal swabs samples were collected using a 20-cm DNA-free sterile swab (Meiruikelin Technology, China) from the nasopharynx mucosa and immediately stored in a dry icebox. Swab samples were kept in a dry ice box and were sent to Chengdu Beisibaier Biotechnology Co., Ltd. (Chengdu, China) for subsequent progress. Every three swab samples were combined into one sample to eliminate the difference induced by individual sampling and reduce sequencing cost by cutting down samples size.

According to Earth Microbiome Project standard protocols (Marotz et al., 2017), DNA was extracted using the MO BIO PowerSoil^TM^ DNA Isolation Kit (MO BIO Laboratories, United States). DNA concentration of all samples was detected by NanoDrop (Thermo Scientific, United States), and the results ranged from 15.2 to 75.4 ng/μl. The qualities of extracted DNA samples were estimated on agarose gel electrophoresis, and only samples that meet the following criteria were used for library construction: (1) DNA concentration was > 15 ng/μl; (2) the total weight of DNA was > 6 μg; and (3) the DNA band that was visualized on agarose gel electrophoresis must be clear and of good quality. Finally, 1 μg DNA of each qualified DNA sample was pooled to an equimolar concentration to construct the DNA libraries (DNA was sheared to 350 bp) using the Illumina DNA Sample Preparation Kit according to the manufacturer’s instructions. Eighteen libraries were constructed, and the amplified libraries were then sequenced on Illumina HiSeq 2500 platform (2 bp × 250 bp). The adaptor contamination was removed using Cutadapt 1.3 (Martin, 2011) with parameters “-o 4 -e 0.1.” Quality control was performed using a sliding window (5 bp bases) in Trimmomatic (Bolger et al., 2014) with the following criteria: (1) cutting once the average quality within the window falls below Q 20; (2) clean reads do not contain any N-bases; (3) trimming is applied to the 3′ end of reads, dropping those reads that were below 50 bp length; (4) only paired-end reads were retained for subsequent analyses. The obtained paired-end clean reads of each sample were performed by *de novo* assembly using Megahit (Li et al., 2010) with the parameter “K-mer ∼ [27, 127]” to contig and scaffold.

### Species and Function Annotation and Analyzation

The species and function annotation procedures were the same as we described in our previous work (Qi et al., 2021). Briefly, the scaffolds/scaftigs sequence of each sample was subjected to BLASTN against bacterial, archaeal, fungal, and viral sequences in the NCBI-NT database (Nucleotide Collection,^1^, v2016-6-19, E value was set to < 0.00001). The “Lowest Common Ancestor” algorithm (Huson et al., 2018) in the MEGAN (MEta Genome Analyzer) software (Huson et al., 2011) was used to distinguish the reference sequence as the last level of a different species before branching and as classification annotation information of the target sequence species. Principal Component Analysis (PCA, Euclidean Distance) (Ramette, 2007) was used to determine whether there was a significant difference between the composition of samples from the same experimental group. A two-tailed *t*-test against the average relative abundance was performed using the SciPy database (Virtanen et al., 2020) in Python software, and the false discovery rate (FDR) was controlled using the Benjamini–Hochberg method (Benjamini and Hochberg, 1995). Only those functional groups with both |Log2 (fold change value)| > 1 and *p* < 0.05 were considered having a significant difference. The species with the significant difference among groups and the top 50 most abundant species were clustered and analyzed using R software. We visualized each sample’s composition structure at each classification level and their relative abundance in heat maps using R software. The linear discriminant analysis (LDA) effect size (LEfSe) (Segata et al., 2011) analysis was performed by submitting the composition spectrum data at the species level to Galaxy online analysis platform^2^. Mothur (Schloss et al., 2009) software was used to calculate Spearman’s grade correlation coefficients (Faust and Raes, 2012) among the 50 most abundant species. The related dominant species with | rho > 0.8| and *p* < 0.01 were used to construct the association network, and the networks were visualized by Cytoscape software (Shannon et al., 2003). The proteins predicted by the MetaGeneMark database (Zhu et al., 2010) were annotated and classified by comparing the protein sequence sets with the Kyoto Encyclopedia of Genes and Genomes (KEGG) Pathway Database (Kanehisa et al., 2004). The non-redundant protein sequence set was uploaded to the KEGG Automatic Annotation Server for functional annotation (parameters: “GENES data set” partially selected “for prokaryotes”; the rest of the parameters were default). The returned annotation results were summarized so that each level’s annotation results and the corresponding abundance information were obtained. Detailed information about the analysis methods we used could be found in our previous paper (Qi et al., 2021).

### Statistical Analysis

The Non-eukaryotic KEGG Orthology (KO) gene’s relative abundances were calculated by normalizing all the KOs of each sample to sum to 1. Observation matrix tables that contain relative abundance information of KOs were used to calculate the Euclidean Distance (Ramette, 2007) based on the UPGMA algorithm (Hua et al., 2017), and the Principal Coordinate Analysis (PCoA) plot was built using the R data analysis package. All figures were drawn by R software and modification by Adobe Illustrator cc (United States). The *p*-value was calculated based on ANOVA and was used to determine whether there was a significant difference in the gene’s abundance between different groups using R software packages and GraphPad Prism 8.0 (United States).

## Results

### Quality Analysis of Sequencing Data

Eighteen libraries were conducted, and a total of 1.23 × 10^11^ bases and 819,054,624 reads were generated. The average base number and reads were 6,825,455,200 and 45,503,035. The average proportion of fuzzy bases (N %) was 0.00423%, and the average percentage of bases with 99.9% accuracy (Q30) was 92.02%. After quality control, 121.38 Mb of high-quality sequencing data were generated for all samples, with averages of 6.21, 7.11, and 6.9 Mb for group A, B, and C samples, respectively. The detailed statistical table of sequencing data is shown in Supplementary Table 1.

**FIGURE 1:**
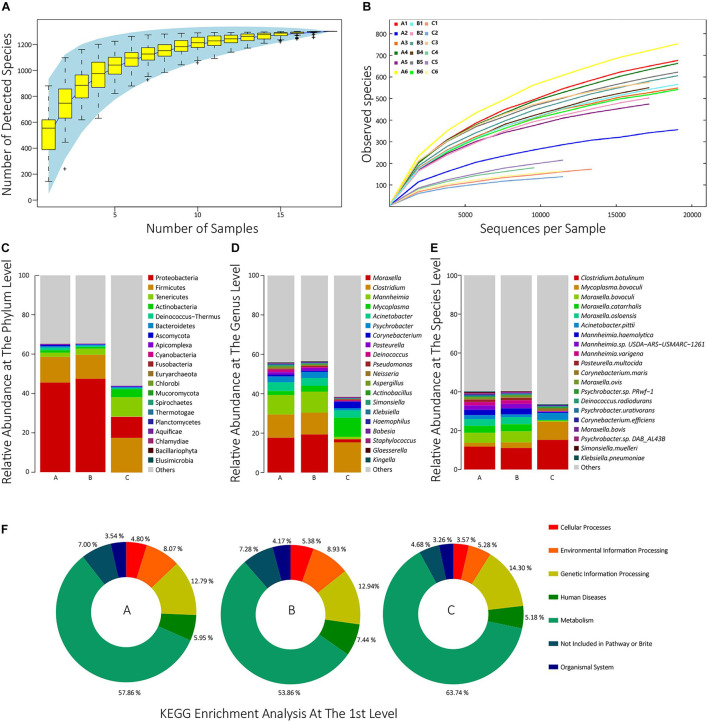
**(A)** The Specaccum species accumulation curve at the species level. The horizontal axis represents the sequencing sample size; the longitudinal axis represents the detected species number at the species level; the blue shadow reflects the curve’s confidence interval; the slope of the curve represents the rate of newly discovered species with expanding samples size. **(B)** Rarefaction curves at the species level of all samples. The horizontal axis represents the number of randomly selected sequences; the longitudinal axis represents the number of detected species in a sequencing depth; the length of the curves represents sequencing depth. If the curve is longer, the sequencing is more deep, and the possibility of detecting new species is higher; the slope of the curve represents the strength of the effect of sample size on the sequencing result, and if the curve is gentler, the sequencing result is more credible for reflecting the composition of the sample. **(C–E)** The top 20 taxon composition with the highest relative abundance of each group at the phylum, genus, and species level, respectively. **(F)** The relative abundances of the first KEGG pathways for each group.

To estimate whether our sample size was big enough to reflect the difference in microbial communities’ composition between samples and estimate microbial communities’ richness, we draw a Specaccum species accumulation curve (Bevilacqua et al., 2018) according to the total number of taxa of each sample at the species level. The curve was flattened when the sample size was 18, which indicated that our sample size was big enough. The curve also implied that the upper limit of the sample species number was approximately 1,300 (Figure 1A). Besides, to determine whether the sequencing depth was deep enough to identify all species, all samples’ rarefaction curves at the species level were drawn. When the sequencing depth reached 20,000 sequences per sample, all the curves trended to flat, which indicated that the sequencing depth was deep enough (Figure 1B).

### Species Composition Analysis

In all 18 samples, a total of 1,301 species from 39 phyla were identified. In group A, the most dominant phylum was Proteobacteria (45.5%), followed by Firmicutes (13.1%), Tenericutes (2.1%), Actinobacteria (1.3%), and Deinococcus-Thermus (1.26%). In group B, the top five phyla were, respectively, Proteobacteria (47.5%), followed by Firmicutes (12.2%), Tenericutes (3.04%), Actinobacteria (0.92%), and Bacteroidetes (0.61%). In group C, they were, respectively, Firmicutes (17.6%), Proteobacteria (10.7%), Tenericutes (9.76%), Actinobacteria (4.18%), and Bacteroidetes (0.52%) (Figure 1C and Supplementary Table 2). In group A, the top five genera were *Moraxella* (17.6%), *Clostridium* (11.8%), *Mannheimia* (9.86%), *Acinetobacter* (4.41%), and *Psychrobacter* (2.95%). In group B, they were, respectively, *Moraxella* (19.3%), *Clostridium* (11.1%), *Mannheimia* (10.6%), *Acinetobacter* (3.92%), and *Mycoplasma* (3.01%). In group C, they were, respectively, *Clostridium* (15.3%), *Mycoplasma* (9.73%), *Acinetobacter* (3.92%), *Corynebacterium* (3.39%), and *Moraxella* (1.61%) (Figure 1D and Supplementary Table 2). The top 30 species with the highest average abundance of each group are, respectively, visualized in Figure 1E. As shown, 1,301 taxa were identified at the species level. In group A, the top five species were, respectively, *Clostridium botulinum* (11.8%), *Moraxella bovoculi* (5.13%), *Moraxella catarrhalis* (3.60%), *Moraxella osloensis* (3.41%), and *M. haemolytica* (2.81%). In group B, they were, respectively, *C. botulinum* (11.1%), *M. bovoculi* (5.68%), *M. catarrhalis* (3.71%), *M. osloensis* (3.56%), and *M. haemolytica* (3.15%). In group C, they were, respectively, *C. botulinum* (15.3%), *Mycoplasma bovoculi* (9.32%), *Acinetobacter pittii* (3.51%), *Corynebacterium maris* (1.40%), and *Psychrobacter* sp. *PRwf-1* (0.586%) (Supplementary Table 2). The detailed species annotation data for each sample are shown in Supplementary Figure 1 and Supplementary Table 3.

### Function Composition Analysis

To analyze the function composition of each sample and each group, the predicted non-redundant protein sets were compared with the KEGG protein database, and a total of 17,120 KEGG orthologs (KOs) and their abundance were identified (Supplementary Table 4). Then, the enrichment analysis of these KOs was performed at the third, second, and first levels among the groups (Supplementary Table 5). Figure 1F visualized the enrichment analysis at the first level for each group. As shown, in group A, the relative abundances of six first-level pathways were, respectively, 57.86% for Metabolism, 12.79% for Genetic Information Processing, 8.07% for Environmental Information Processing, 5.95% for Human Diseases, 4.80% for Cellular Processes, and 3.53% for Organismal Systems. In group B, the relative abundances were 53.9% for Metabolism, 12.9% for Genetic Information Processing, 8.93% for Environmental Information Processing, 7.44% for Human Diseases, 5.38% for Cellular Processes, and 4.17% for Organismal Systems. In group C, they were 63.7% for Metabolism, 14.3% for Genetic Information Processing, 5.28% for Environmental Information Processing, 5.18% for Human Diseases, 3.57% for Cellular Processes, and 3.26% for Organismal Systems.

### Analysis of Interconnection Network of Dominant Species

**FIGURE 2:**
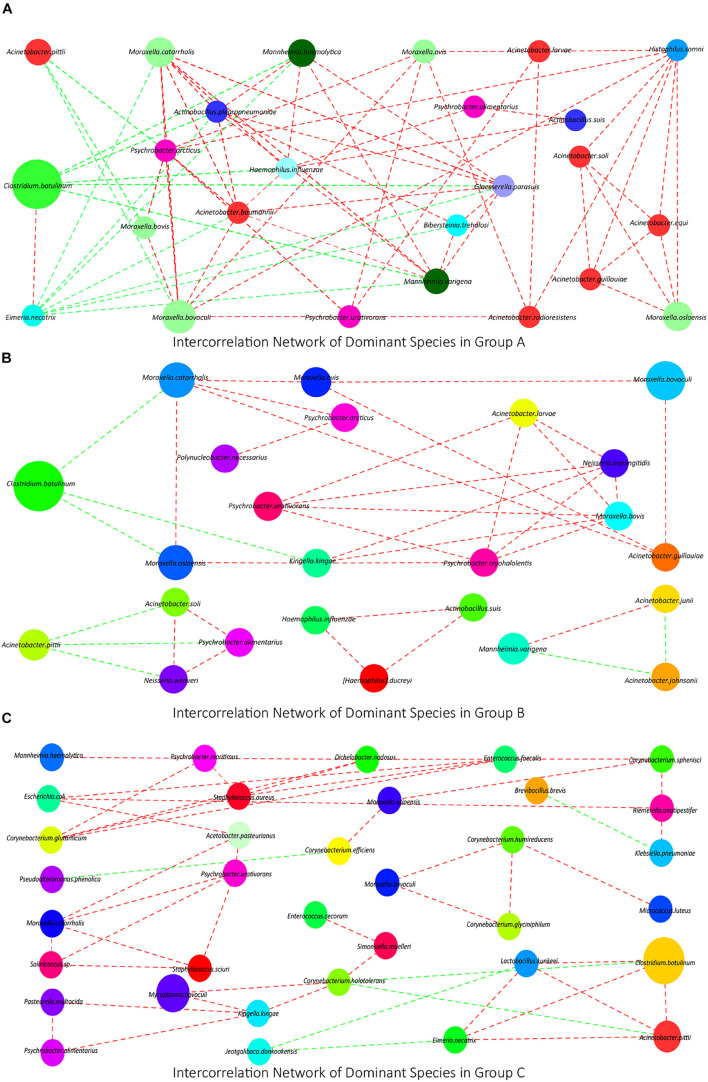
**(A–C)** The interconnection networks of the top 50 species in groups A, B, and C, respectively. Red lines mean positive correlations, green lines mean negative correlations, and the size of circles represents the relative abundance of the corresponding species.

To investigate the interconnection of the dominant species in each group, we calculated the correlation coefficient of Spearman grade of the top 50 species in each group based on the species abundance composition and visualized the conducted co-correlation networks using Cytoscape. In group A, a network containing 25 kinds of species was obtained. In this network, 22 of them had positive interconnection with each other, except *A. pittii*, *C. botulinum*, and *Eimeria necatrix*, which had negative interconnections with other (Figure 2A). In group B, a smaller network containing 14 kinds of species and three micro-networks were obtained. *C. botulinum* and *A. pittii* also had negative interconnections with others (Figure 2B). In group C, two dominant networks and a micro-network were obtained, which contained less *Moraxella* or *Acinetobacter* (Figure 2C).

### Difference Analysis in Species Composition

**FIGURE 3:**
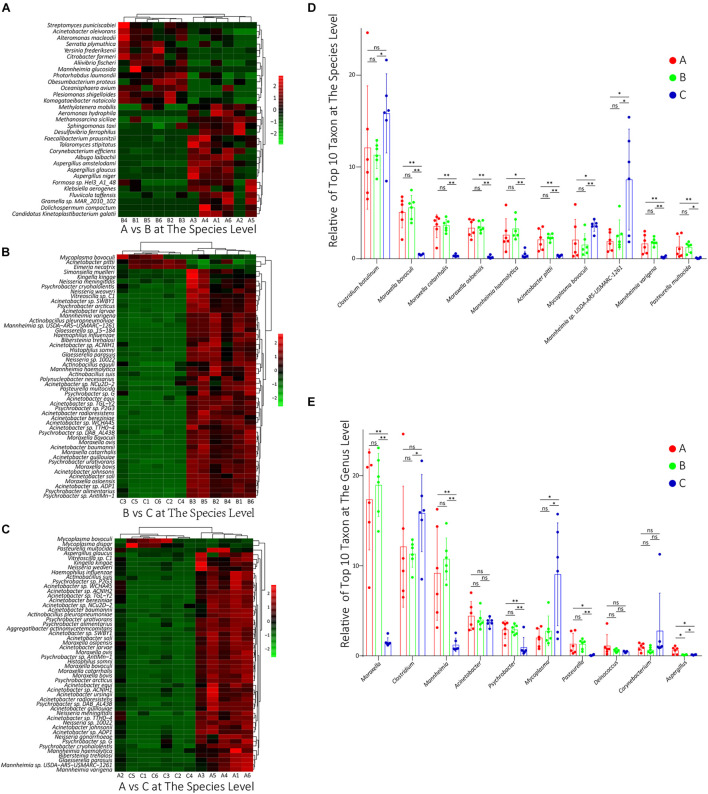
**(A–C)** The results of cluster analysis on the top 50 taxa with significant differences between groups A and B, between groups B and C, and between groups A and C, respectively, at the species level. The red box denotes that the relative abundance was higher, and the green box indicates that the relative abundance was lower. **(D,E)** The relative abundance of these top 10 species and genus, respectively, in all groups. ns: non-significant; ^∗^0.01 < *p* < 0.05; ^∗∗^*p* < 0.01.

To compare the differences in species composition among groups, firstly, we compared the abundance differences of each taxon among the three groups, and the statistical test was used to evaluate whether the differences were significant. The statistics data of abundance difference results are shown in Table 1. The detailed statistics data are shown in Supplementary Table 6. Then, using the R script, we conducted a cluster analysis on the top 50 taxa with significant differences among groups at the species level, and the results were shown in heat map form (Figures 3A–C). As shown, there were dominant populations for both groups A and B, but the relative abundance of populations in group C was almost significantly lower than that in group A or B, which indicated that the richness of the microflora of group C was lower than groups A and B. The cluster analysis results at the genus level are shown in Supplementary Figure 2, showing a similar phenotype. Considering that most of these significantly changed species had an extremely low relative abundance, we counted the significance of differences in those species and genus, which possessed the top 10 relative abundance between groups. At the species level, the differences in the relative abundance of the top 10 species were all non-significant between groups A and B, but between groups B and C and between groups A and C, the differences were almost all significant (Figure 3D). At the genus level, except *Aspergillus*, the relative abundances of all other nine genera had non-significant changes between groups A and B. However, between groups B and C and between groups A and C, the relative abundances of *Moraxella*, *Clostridium*, *Mannheimia*, and *Mycoplasma* were significantly changed (Figure 3E).

**TABLE 1:** Statistical table of abundance difference analysis.

Group	Phylum	Genus	Species
A vs. B	5	31	48
B vs. C	13	190	378
A vs. C	11	166	330

*A vs. B denotes that the comparison was performed in groups A and B; the number means the size of phylum/genus/species with a significant difference.*

### Difference Analysis in Function Composition

To compare the differences in function composition among groups, we compared the abundance differences of each functional taxon among the three groups. The *p*-value and FDR were used to evaluate whether the differences were significant, and it was found that 64, 905, and 1,008 KOs were significantly changed between groups A and B, B and C, and A and C, respectively. The detailed data are shown in Supplementary Table 7. We also conducted a cluster analysis on the top 50 taxa with significant differences in KOs among groups, and the results are shown in Supplementary Figures 2D–F. Then, we performed a KEGG pathway enrichment analysis for the significantly changed KOs among groups, and the results are shown in Table 2. The KOs related to the Immune system, Immune diseases, and Infectious diseases were significantly enriched (*p* < 0.05) in group A against group B. The KOs related to Lipid metabolism and Carbohydrate metabolism were significantly enriched in group B against group C (*p* < 0.05). The KOs related to the Amino acid metabolism and Glycan biosynthesis and metabolism were significantly enriched in group C against group B (*p* < 0.05). The KOs related to Ribosome and Starch and sucrose metabolism were significantly enriched in group C against group A (*p* < 0.05).

**TABLE 2:**
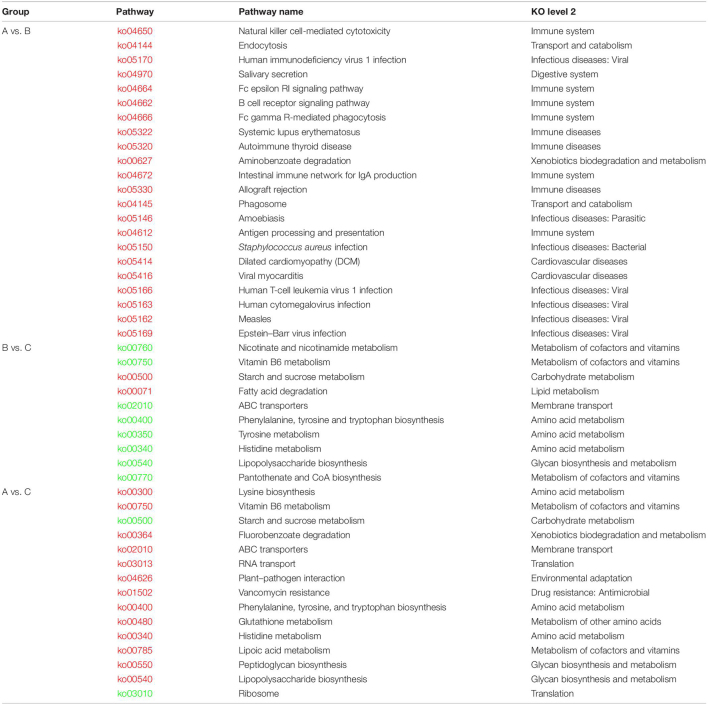
Results of KEGG enrichment analysis on significantly changed KOs among groups.

*Red pathways mean that the pathway was enriched in the front group; green pathways mean that the pathway was enriched in the back group.*

### Diversity Analysis

**FIGURE 4:**
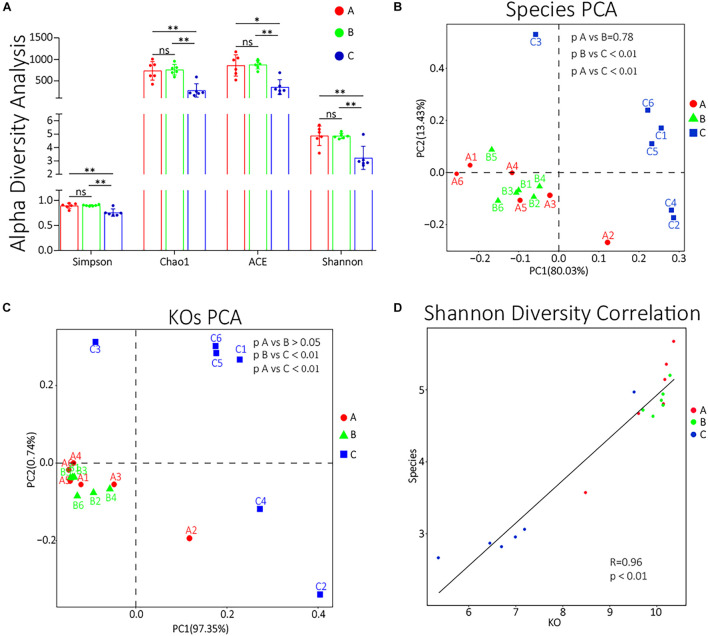
**(A)** α diversity indexes of each group. ns, non-significant; ^∗^0.01 < *p* < 0.05; ^∗∗^*p* < 0.01. **(B,C)** The species PCA and KOs PCA, respectively, of each sample. **(D)** The Pearson correlation analysis of the Shannon diversity indexes of KOs and species. The abscissa represents the Shannon diversity index corresponding to the KO abundance spectrum of each sample, and the ordinate represents the Shannon index corresponding to the composition spectrum of each sample at the species level. The straight line represents the fitting results by Pearson correlation analysis.

To compare the α diversity of each group, using QIIME software, we calculated the diversity indexes of Chao l, ACE, Simpson, and Shannon based on the abundance spectrum of bottom functional groups and species groups. All these four indexes of group C were significantly lower (*p* < 0.05) than groups A and B (Figure 4A). The detailed statistical data of α diversity indexes are shown in Supplementary Table 8. The unsupervised β diversity analysis was also performed. PCA of species and KOs were performed, and the results are shown in Figures 4B,C, respectively. Both species and KO PCA results showed no significant difference between groups A and B (*p* > 0.05) and that the differences between groups B and C or between groups A and C were highly significant (*p* < 0.01). To further evaluate whether the patterns of differences in the functional and species levels among groups are correlated, we also performed a Pearson correlation analysis to the Shannon indexes of species and function (Figure 4D). The results showed that the function difference among groups was highly correlated with the species difference (*R* = 0.96, *p* < 0.01).

## Discussion

In the present study, the dynamics of nasopharyngeal microflora of 3 days of transportation and 7 days of adaptive feeding was studied by a metagenomic shotgun sequencing approach. We analyzed the composition of a taxon at the species and genus level, the composition of function at different KO levels, and the α diversities for both samples and groups. Then, to investigate whether the influence of transportation and adaptive feeding on the nasopharynx microflora was significant, we calculated the difference based on the species and functional composition using multiple statistical methods. The structure of beef cattle’s nasopharyngeal microflora has been clarified in previous work (Zeineldin et al., 2017; Amat et al., 2019; Mcmullen et al., 2020). Here, we focused on the dynamic variation induced by transportation or placement.

### Tracking Sampling Without Interference and Metagenomic Shotgun Sequencing Allow Us to Analyze the Dynamics More Precisely

In the experiment, we never take part in the transportation or placement, and the farmer determined all the processes as he usually did. This study’s transportation and placement procedures might represent a widely used process in Southwest China, including choosing the auction market and calves, the uninterrupted vehicle transportation, and the brown sugar and ginger in the water during the placement. Holman et al. (2017) performed a very similar experiment in which they specified the source of cattle and excluded cattle with specific pathogens, which might affect its results. Hence, the dynamics we observed could be more similar to the actual situation. Besides, the sequencing approach we used was a recently developed next-generation sequencing approach with higher accuracy than 16S rRNA sequencing technology. For example, Timsit and colleagues observed 963 taxa in the nasopharynx sample (Mcmullen et al., 2018) while identifying 1,301 species. For now, metagenomic shotgun sequencing has been widely used to explore the respiratory system (Qi et al., 2021) or the digestive system microorganism communities (Wang et al., 2015). In our study, the taxonomic level we identified even reached subspecies, which allowed us to analyze the composition and dynamics of the microorganism community more precisely. With these advantages, our results could be a more valuable reference.

### Three Days of Transportation From an Auction Market to a Feedlot Did Not Significantly Change the Calves’ Nasopharyngeal Microflora Community

In species PCA and KOs PCA (Figures 4B,C), there was no significant difference observed between groups A and B, and the α diversity of groups A and B had no significant difference either (Figure 4A). Besides, there were no significant differences in the relative abundances of the top 10 species or genus between groups A and B (Figures 3D,E). Therefore, our results generally showed that 3 days of transportation had no significant effect on the structure or function of the nasopharyngeal microorganism community of beef calves. The same conclusion was also shared by some previous works. For example, Ribble et al. (1995) found that the transport distance did not affect the risk of developing fatal fibrinous pneumonia by observing the pathological features of 45,243 spring-born steer calves purchased from auction markets. Timsit’s team also found that the transportation from a feedlot to another feedlot directly or 24 h of co-mingling at an auction market did not significantly change cattle’s nasopharyngeal microbial community (Stroebel et al., 2018). However, it cannot be ignored that we did not know how long these calves had been in this market, which means that we did not have the baseline information of the nasopharyngeal microbial community. It is unclear whether long-term exposure in the auction market has a significant effect on the microbial community, because the structure and diversity could have already been significantly affected before transportation, which weakened the influence of transportation and resulted in the non-significance. The difference of baseline might explain why some other works found that transportation significantly altered the composition and structure of the microbial community (Holman et al., 2017; Pratelli et al., 2021). The baseline they used was in the sourced feedlot, and the calves were healthy, while the baseline we used (group A) was in the market, and the calves were probably not as healthy as they were, and the nasopharynx microbiota might have been significantly altered already. However, this hypothesis needs to be verified. In groups A and B, we noticed a high abundance of *Moraxella*, *Mannheimia*, and *Acinetobacter*, which were considered potential pathogenic bacteria of BRD (Holman et al., 2015; Zeineldin et al., 2017) (Figures 3D,E), forming an interconnection network (Figures 2A,B), and this microbial community structure was similar to those cattle with BRD (Holman et al., 2015; Zeineldin et al., 2017). This structure might be a certification that the microbial community of these calves were already affected before transportation. In short, based on our results, 3 days of transportation from an auction market to a feedlot did not significantly change the calves’ nasopharyngeal microflora community, probably because these calves’ nasopharynx microbiota was already significantly altered in the auction market.

### Transportation Affected the Interaction Between the Nasopharyngeal Microbial Community and the Host

Though our results showed that transportation had no significant effect on microbial community, some of these changes in species and KOs might provide us detailed information during the transport. Firstly, we noticed that the relative abundance of *M. haemolytica*, *Pasteurella multocida*, *Haemophilus somni*, *M. bovoculi*, etc., which are widely considered BRD pathogens (Holman et al., 2015; Amat et al., 2019), increased after transportation (Figure 3D), though the increase was non-significant. Besides, the relative abundance of *C. botulinum* decreased, though the decrease was non-significant either (Figure 3D). *C. botulinum* is the most dominant species in cattle’s nasopharynx (Mcmullen et al., 2020; Qi et al., 2021), which negatively correlated with most of the other dominant species in the network consisting of many BRD pathogens (Figure 2A). Furthermore, the interconnection among the top 50 species is more tight in group A than in group B (Figures 2A,B). Hence, from the perspective of species, the decrease of *C. botulinum* and the increase of BRD pathogens implied that if the exposure in the market altered the baseline, the transportation probably increased the risk of BRD. From the perspective of KOs, in the KEGG enrichment analysis, we noticed that many KOs that associated with the Immune system, Immune diseases, and Infectious diseases were enriched in group A compared with group B (Table 2), which implied that the microbial community after transportation had less connection with the host’s immune system. The balance between the microflora and host was broken up by the 3 days of transportation, which also means the higher risk of BRD (Zeineldin et al., 2019). Considering these results, though the 3 days of transportation did not significantly change the composition or structure of the microbial community, it affected the interaction between the nasopharyngeal microbial community and the host and increased the risk of BRD.

### Proper Adaptive Placement Is Necessary for the Health of Transported Calf

In the present study, the adaptive feeding with brown sugar and ginger after transportation significantly altered the diversity, structure, and composition of the transported calf’s nasopharynx microbial community. In the PCA, the species and KOs of group C were both significantly (*p* < 0.05) different from groups A and B (Figures 4B,C). The α diversity indexes of group C were extremely different (*p* < 0.01) from groups A and B (Figure 4A). The relative abundances of the top 10 species and genus in group C were almost all significantly different (*p* < 0.05) from groups A and B (Figures 3D,E). The size of significantly changed species/genus/phylum between groups A and C or between groups B and C was much bigger than between groups A and B (Table 1). All these results indicated that an adaptive placement significantly affected the microbial community, supported by previous works (Hall et al., 2017; Schuetze et al., 2017; Amat et al., 2019). However, the consequence of this alteration seemed to be incomprehensible. Normally, the α diversity is positively related to the health of the cattle’s respiratory system (Man et al., 2017; Zeineldin et al., 2019), but our results showed that the α diversity was extremely (*p* < 0.01) decreased after 7 days of placement (Figure 4A), which was contrary to previous work (Hall et al., 2017; Pratelli et al., 2021) and seemed to be harmful to the transported calves. We concluded the following reasons for the significant decrease in α diversity. Firstly, ginger was found to possess antimicrobial activity (Noori et al., 2018; Beristain-Bauza et al., 2019), so 3 days of ginger supply might inhibit the growth of some high abundant microorganisms, most of which were potential BRD-related pathogens. We noticed that the significantly decreased species (*M. bovoculi*, *M. catarrhalis*, *M. osloensis*, *M. haemolytica*, *P. multocida*, etc.) and genus (*Moraxella*, *Mannheimia*, and *Pasteurella*) were widely accepted to be related to the progress of BRD (Holman et al., 2015; Cirone et al., 2019; Mcmullen et al., 2019). Secondly, the recovered immune function inhibited the growth of pathogens. During the placement, calves were released from transport stress and were free to drink and eat, which supplied the deficiency in energy and enhanced the immune function (Earley et al., 2017; Qi et al., 2021). Besides, the relative abundance of Peptidoglycan biosynthesis (Bouhss et al., 2008) and Lipopolysaccharide biosynthesis (Heinrichs et al., 1998) in group A was enriched, implying that the synthesis level of the cell wall in group C was lower than that in group A and that the renewal of bacteria was inhibited. Furthermore, brown sugar is widely used in Chinese livestock’s breeding industry, such as chicken and calf, which is thought to quickly replenish energy and contribute to the recovery of calf immune function (State Pharmacopoeia Commission of the PRC, 2005). However, there are few studies regarding the beneficial influence of brown sugar on the calf and these default roles of brown sugar are not confirmed and need to be investigated. Thirdly, as discussed in previous work (Qi et al., 2021), because of the supply of fodder, the absolute abundance of oral bacteria significantly increased, and they would compete for living resources (ecological locus, energy, etc.) with pathogens in the nasopharynx, which resulted in the decrease of pathogens. Moreover, the interconnection among group C was tighter and diversified. Hence, although the α diversity of the microbial community was decreased, the risk of BRD was also decreased. We speculated that the development of symbiotic bacteria needs to remove the existing pathogens, and 7 days of placement of brown sugar and ginger supply were not long enough to reestablish the balance between host and microbiota. This hypothesis explained why the α diversity of our microbial community decreased while it was increased in previous work (Hall et al., 2017; Pratelli et al., 2021) and enlightened us that this placement might not be appropriate. Nevertheless, further evidence is still needed.

## Conclusion

In summary, after long-distance transportation, in the nasopharynx, the most abundant phylum was Proteobacteria (45.5%), followed by Firmicutes (13.1%), and the most dominant genus was *Moraxella* (17.6%), followed by *Clostridium* (11.8%) and *Mannheimia* (9.86%). Like previous works, the nasopharyngeal microbial community of transported calves was of a great abundance of potential BRD-related pathogens, which was closed to the cattle diagnosed with BRD. Probably because of the difference in baseline, our results showed that 3 days of transportation had no significant effect on the nasopharyngeal microbial community, which some other researchers also observed. The placement of brown sugar and ginger significantly decreased the relative abundance of those potential BRD-related pathogens and altered the functional composition of the microbial community, which confirmed that the adaptive placement had a stronger influence on the calf nasopharynx microbiome than transportation itself and indicated that the risk of BRD was decreased and that proper adaptive placement was critical for the transported calf respiratory system’s health. Interestingly, the α diversity of the microbial community was significantly decreased after the placement, which is contrary to previous work. The reason for this phenotype was hypothesized, but it needs to be further verified. Because of the lack of physiological data, sample size limitation, and the lack of baseline information, further experiments are still needed.

## Data Availability Statement

The datasets presented in this study can be found in online repositories. The names of the repository/repositories and accession number(s) can be found below: https://www.ncbi.nlm.nih.gov/bioproject/PRJNA724913/.

## Ethics Statement

The animal study was reviewed and approved by Ethics Committee of Sichuan Agricultural University (Chengdu, China). Written informed consent was obtained from the owners for the participation of their animals in this study.

## Author Contributions

ZcZ and ZW designed the experiment. YC and JQ wrote the manuscript. All authors reviewed and revised the manuscript.

## Conflict of Interest

The authors declare that the research was conducted in the absence of any commercial or financial relationships that could be construed as a potential conflict of interest.

## Publisher’s Note

All claims expressed in this article are solely those of the authors and do not necessarily represent those of their affiliated organizations, or those of the publisher, the editors and the reviewers. Any product that may be evaluated in this article, or claim that may be made by its manufacturer, is not guaranteed or endorsed by the publisher.

## Footnotes

1
ftp://ftp.ncbi.bih.gov/blast/db/

2
huttenhower.sph.harvard.edu/galaxy/

## Supplementary Material

Supplementary Figure 1 **(A–C)** The top 20 taxon composition with the highest relative abundance of each sample at the phylum, genus, and species level, respectively.

Supplementary Figure 2 **(A–C)** The results of cluster analysis on the top 50 taxa with significant differences between group A and B, B and C, and A and C, respectively, at the genus level. **(D–F)** The results of cluster analysis on the top 50 KOs with significant differences between groups A and B, B and C, and A and C, respectively, at the 3rd level. The red box represents that the relative abundance was higher, the green box represents that the relative abundance was lower.

Supplementary Table 1 Statistical table of raw data and clean data.

Supplementary Table 2 The species annotation data of each group at the phylum, genus, and species level, respectively.

Supplementary Table 3 The detailed species annotation data for each sample.

Supplementary Table 4 The detailed information of 17,120 KEGG orthologs (KOs) and their abundance.

Supplementary Table 5 The KO enrichment analysis among groups at the 1st, 2nd, and 3rd, respectively.

Supplementary Table 6 The detailed statistics data of abundance differences among groups at phylum/genus/species level, respectively.

Supplementary Table 7 The detailed statistics data of relative abundance differences in KOs among groups.

Supplementary Table 8 Statistical table of alpha diversity for each sample.
