# Supplementary figures and images for: Metagenomics Reveals That Proper Placement After Long-Distance Transportation Significantly Affects Calf Nasopharyngeal Microbiota and Is Critical for the Prevention of Respiratory Diseases
Source: Front Microbiol. 2021 Sep 20;12:700704. doi: 10.3389/fmicb.2021.700704 (PMC8488368; doi:10.3389/fmicb.2021.700704)

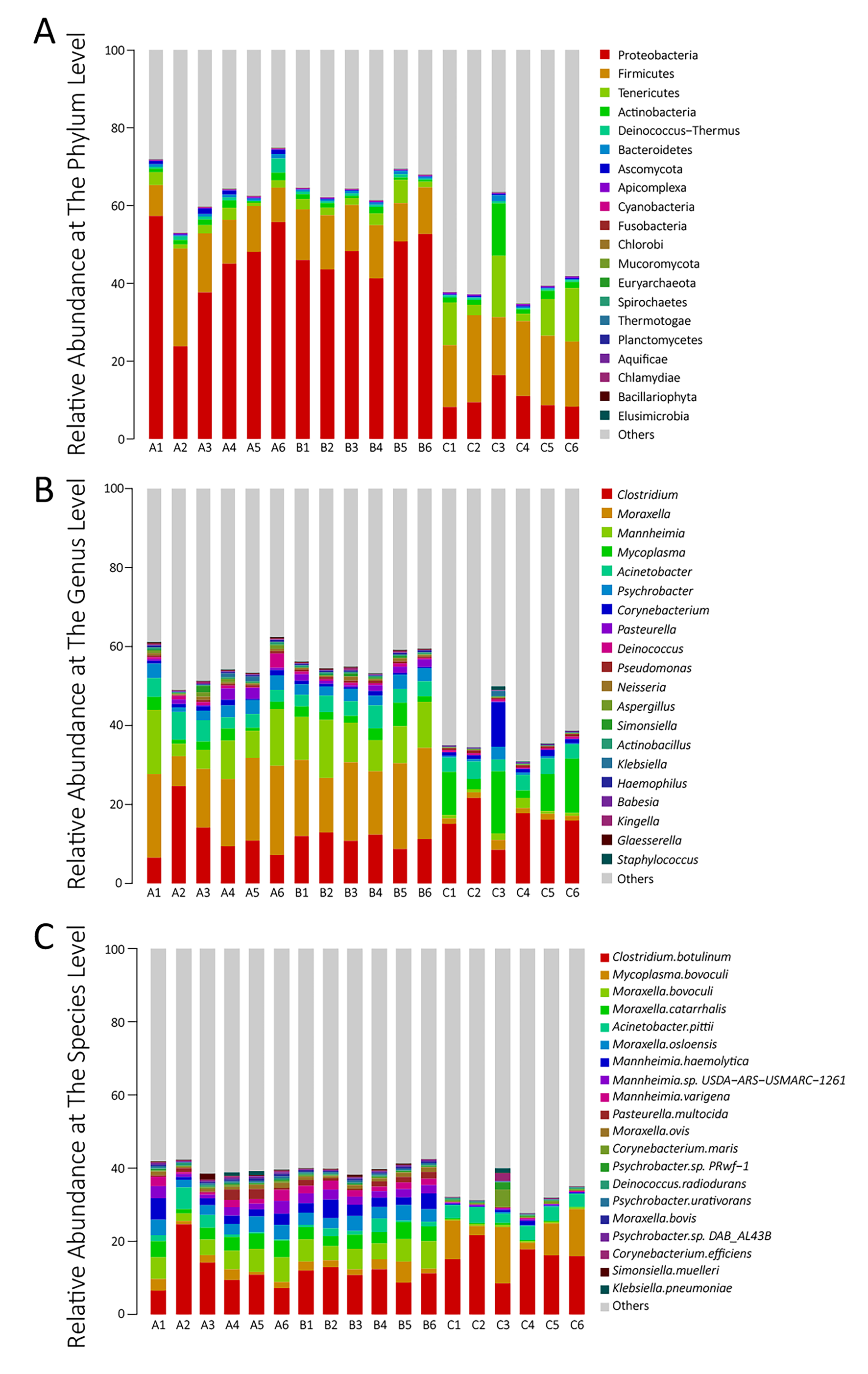

Supplement: Supplementary Figure 1 — (A–C) The top 20 taxon composition with the highest relative abundance of each sample at the phylum, genus, and species level, respectively. [file Image_1.TIF]

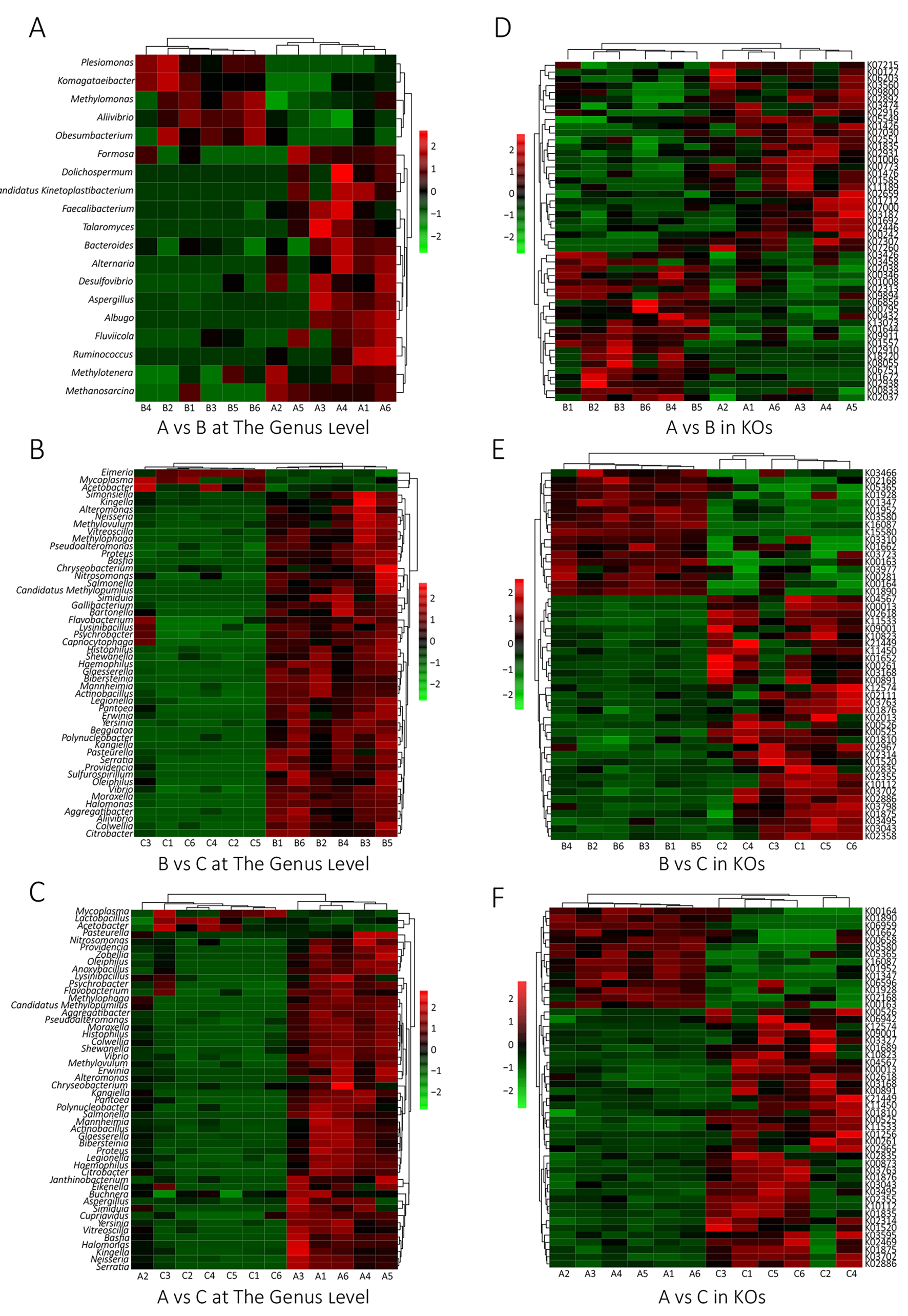

Supplement: Supplementary Figure 2 — (A–C) The results of cluster analysis on the top 50 taxa with significant differences between group A and B, B and C, and A and C, respectively, at the genus level. (D–F) The results of cluster analysis on the top 50 KOs with significant differences between groups A and B, B and C, and A and C, respectively, at the 3rd level. The red box represents that the relative abundance was higher, the green box represents that the relative abundance was lower. [file Image_2.TIF]
