# Supplementary material for: Metagenomics Reveals That Proper Placement After Long-Distance Transportation Significantly Affects Calf Nasopharyngeal Microbiota and Is Critical for the Prevention of Respiratory Diseases
Source: Front Microbiol. 2021 Sep 20;12:700704. doi: 10.3389/fmicb.2021.700704 (PMC8488368; doi:10.3389/fmicb.2021.700704)
Supplement: Supplementary Table 1 — Statistical table of raw data and clean data. [file Table_1.DOCX]

| Sample | Reads Number | Bases Number | N % | GC（%） | Q20（%） | Q30（%） |
| --- | --- | --- | --- | --- | --- | --- |
| A1 | 40975134 | 6146270100 | 0.00582 | 46.85 | 97.06 | 92.34 |
| A2 | 50200138 | 7530020700 | 0.00184 | 50.4 | 96.34 | 91.15 |
| A3 | 46265766 | 6939864900 | 0.00578 | 46.19 | 97.08 | 92.39 |
| A4 | 35370492 | 5305573800 | 0.00587 | 47.37 | 96.63 | 91.43 |
| A5 | 37184872 | 5577730800 | 0.00584 | 45.47 | 96.62 | 91.31 |
| A6 | 41882322 | 6282348300 | 0.00584 | 47.04 | 97.19 | 92.62 |
| B1 | 46510192 | 6976528800 | 0.00575 | 45.21 | 96.25 | 90.26 |
| B2 | 42166796 | 6325019400 | 0.00228 | 45.27 | 97.13 | 92.44 |
| B3 | 46266192 | 6939928800 | 0.00230 | 45.15 | 97.33 | 92.84 |
| B4 | 52134738 | 7820210700 | 0.00231 | 45.35 | 97.36 | 92.90 |
| B5 | 58574706 | 8786205900 | 0.00232 | 44.97 | 97.01 | 92.10 |
| B6 | 42280518 | 6342077700 | 0.00047 | 45.53 | 97.32 | 92.89 |
| C1 | 52406310 | 7860946500 | 0.00580 | 44.75 | 96.54 | 91.13 |
| C2 | 57011842 | 8551776300 | 0.00047 | 44.82 | 96.95 | 92.07 |
| C3 | 40674774 | 6101216100 | 0.00588 | 44.77 | 97.30 | 92.77 |
| C4 | 38777258 | 5816588700 | 0.00588 | 44.71 | 96.68 | 91.44 |
| C5 | 47048114 | 7057217100 | 0.00591 | 44.32 | 96.98 | 92.07 |
| C6 | 43324460 | 6498669000 | 0.00583 | 44.63 | 97.03 | 92.18 |
| Total | 819054624 | 1.22858E+11 |  |  |  |  |
| Average | 45503035 | 6825455200 | 0.00423 | 45.71 | 96.93 | 92.02 |

Statistical table of raw data

N %: the proportion of fuzzy bases to the total number of bases; GC %: the ratio of G base and C base to the total number of bases; Q20 (%) and Q30 (%): the percentage of bases with 99% and 99.9% accuracy in the total number of bases, respectively.

Statistical table of clean data

| Sample | HQ Reads (%) | HQ Data (bp) | HQ Data (%) |
| --- | --- | --- | --- |
| A1 | 92.34 | 6069374760 | 98.75 |
| A2 | 91.15 | 7419847858 | 98.54 |
| A3 | 92.39 | 6848810558 | 98.69 |
| A4 | 91.43 | 5228532535 | 98.55 |
| A5 | 91.31 | 5509052807 | 98.77 |
| A6 | 92.62 | 6198056213 | 98.66 |
| B1 | 90.26 | 6884611630 | 98.68 |
| B2 | 92.44 | 6248654803 | 98.79 |
| B3 | 92.84 | 6865077461 | 98.92 |
| B4 | 92.90 | 7740726965 | 98.98 |
| B5 | 92.10 | 8688950139 | 98.89 |
| B6 | 92.89 | 6273808777 | 98.92 |
| C1 | 91.13 | 7759422307 | 98.71 |
| C2 | 92.07 | 8449204866 | 98.80 |
| C3 | 92.77 | 6041742344 | 99.03 |
| C4 | 91.44 | 5745490590 | 98.78 |
| C5 | 92.07 | 6980452885 | 98.91 |
| C6 | 92.18 | 6429226980 | 98.93 |
| Average | 92.02 | 6743391360 | 98.79 |

HQ Reads (%): percentage of high-quality sequences in the number of original sequences; HQ Data (bp): total base number of high-quality sequences; HQ Data (%): the percentage of the total number of bases in the high-quality sequence to the total number of bases in the original sequence.
