# Supplementary material for: Metagenomics Reveals That Proper Placement After Long-Distance Transportation Significantly Affects Calf Nasopharyngeal Microbiota and Is Critical for the Prevention of Respiratory Diseases
Source: Front Microbiol. 2021 Sep 20;12:700704. doi: 10.3389/fmicb.2021.700704 (PMC8488368; doi:10.3389/fmicb.2021.700704)
Supplement: Supplementary Table 8 — Statistical table of alpha diversity for each sample. [file Table_8.DOCX]

| Sample | Alpha Diversity | | | |
| --- | --- | --- | --- | --- |
|  | Simpson | Chao1 | ACE | Shannon |
| A1 | 0.931574134 | 878 | 989.1990099 | 5.3670882 |
| A2 | 0.796106079 | 425.1212121 | 522.5175852 | 3.58325869 |
| A3 | 0.873832775 | 619.9285714 | 769.3217083 | 4.677017357 |
| A4 | 0.90642259 | 859.9607843 | 1032.79777363 | 5.1469776 |
| A5 | 0.892973747 | 622.6071429 | 673.5651874 | 4.81405224 |
| A6 | 0.94133323 | 1009.142857 | 1177.244578 | 5.685214831 |
| Average | 0.890373759 | 735.793428 | 826.3696137 | 4.87893482 |
| B1 | 0.894388183 | 794. 757575758 | 897.9120334 | 4.79822445503 |
| B2 | 0.882636609 | 593.4285714 | 714.5799839 | 4.635790576 |
| B3 | 0.897936206 | 808.5 | 945.693903 | 4.947296144 |
| B4 | 0.881799187 | 714.5849057 | 873.2365733 | 4.722545061 |
| B5 | 0.919404445 | 957 | 998.4401534 | 5.20885202277 |
| B6 | 0.906814523 | 693.125 | 821.3232329 | 4.857441338 |
| Average | 0.897163192 | 753.3276954 | 875.1976466 | 4.79076828 |
| C1 | 0.727772266 | 222.125 | 277.232567362 | 2.826277065 |
| C2 | 0.703868407 | 163.3076923 | 206.7375399 | 2.673640736 |
| C3 | 0.89409007724 7 | 590.1081081 | 690.302224 | 4.979368594 |
| C4 | 0. 705427865781 | 230 | 309.8966106 | 2.872447957 |
| C5 | 0.746916462 | 255.625 | 381.1078223 | 3.063217739 |
| C6 | 0.751131061 | 208 | 270.1300483 | 2.957474128 |
| Average | 0.732422049 | 278.1943001 | 371.634849 | 3.228737703 |
| p A-B | 0.765826959 | 0.879134895 | 0.677989552 | 0.821372144 |
| p B-C | 4.71852E-07 | 0.000488148 | 0.000298503 | 0.008058103 |
| p A-C | 0.000493087 | 0.001801242 | 0.013190522 | 0.005182882 |

Statistical table of alpha diversity for each sample

p A-B, p B-C and p A-C: the p-value between A and B, B and C, and A and C, respectively.
